# Endotracheal sialolipoma: A rare case report

**DOI:** 10.1097/MD.0000000000046906

**Published:** 2026-01-02

**Authors:** Ying Xu, Zhi-Bin Gao, Jian-Feng Chu, Qiong-Nan Shen

**Affiliations:** a Department of Pathology, Yuyao People’s Hospital, Yuyao, China.

**Keywords:** case report, endotracheal, sialolipoma, treatment

## Abstract

**Rationale:**

Sialolipomas arising in the tracheobronchial tree are exceedingly rare. To date, only 6 cases of tracheobronchial sialolipoma have been documented in the English medical literature. A comprehensive understanding of the characteristics of this tumor is vital for accurate diagnosis and the development of effective treatment strategies. This study seeks to elucidate its features through a detailed case report and literature synthesis, aims to prevent diagnostic pitfalls and guide optimal treatment for this exceedingly rare tumor.

**Patient concerns:**

A 28-year-old male was referred to Yuyao People’s Hospital in December 2024, due to cough and shortness of breath for nearly 1 year.

**Diagnoses:**

Non-contrast chest computed tomography (CT) revealed a nodular lesion in the trachea. Bronchoscopy showed a neoplasm located approximately 10 mm above the tracheal carina, causing over 70% luminal stenosis. Pathological analysis of the surgical specimen led to a diagnosis of sialolipoma.

**Interventions:**

The patient underwent a lesion resection procedure via rigid bronchoscopy.

**Outcomes:**

The patient recovered well. A follow-up CT scan at 7 months after tumor removal showed no evidence of recurrence.

**Lessons:**

Endotracheal sialolipoma is a rare benign tumor. CT and magnetic resonance imaging are valuable tools for diagnosis (CT Hounsfield units suggest fatty components in the tumor). The primary treatment is endoscopic tumor resection. The prognosis is favorable after complete excision.

## 1. Introduction

Sialolipoma is very rare, and is considered to be a variant of salivary gland lipoma. It was first described and reported by Nagao et al in 2001.^[1]^ This tumor has been classified as a benign soft-tissue tumor in the 2017 World Health Organization classification of head and neck tumors. Based on the benign nature of the salivary gland components in the tumor, the recent WHO classification (2022) does not recommend using the term “adenolipoma.” Sialolipoma is composed of salivary gland and mature adipose tissue with clear boundaries. Typically, sialolipomas occur in both major and minor salivary glands, with the majority of cases observed in the parotid gland.^[2]^ Sialolipoma in tracheobronchial tree is extremely rare. We searched the literature on PubMed up to February 2025, using the terms “sialolipoma,” “adenolipoma,” “tracheal,” “tracheobronchial,” and “bronchus.” Only 6 cases have been published, 2 of which were diagnosed as adenolipoma.^[3–8]^ Here we report a case of endotracheal sialolipoma.

No formal statistical analysis was performed due to the single-patient design. The literature review was narrative, with descriptive summarization of previously reported cases.

## 2. Case report

A 28-year-old male was referred to the Yuyao People’s Hospital in December 2024, due to cough and shortness of breath for nearly 1 year. Physical examination showed inspiratory stridor.

**Figure 1.**
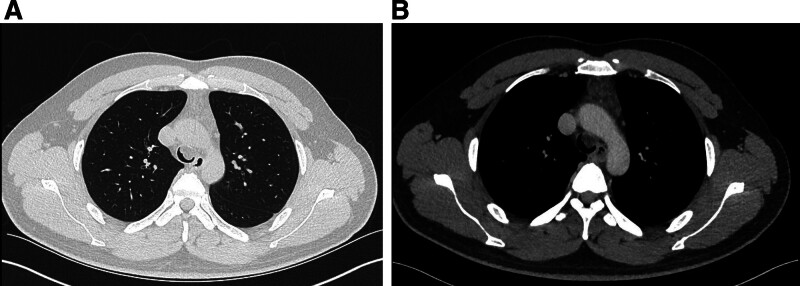
Contrast-enhanced chest CT. (A, B) Above the carina of trachea a nodular shadow is observed in the trachea adjacent to the anterior wall, protruding inward of the cavity.

Non-contrast chest computed tomography (CT) revealed a nodule lesion in the trachea. For further treatment, the patient was hospitalized, and underwent a contrast-enhanced chest CT. The CT showed a tracheal mass with fat density (approximately–49 HU). The lesion exhibited clear boundaries and a lobulated shape (Fig. 1A, B). After contrast agent injection, the lesion showed enhancement, indicating the presence of nonfatty components. Magnetic resonance imaging (MRI) can help characterize fat and nonfat components. It was not performed in this case.

**Figure 2.**
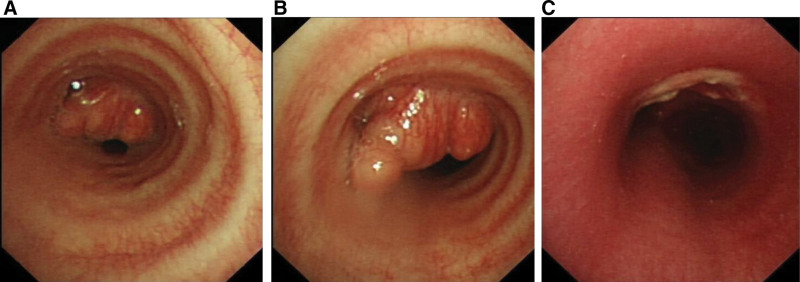
Bronchoscopy images. (A, B) The middle and upper part of the trachea were unobstructed, and a neoplasm was observed at a distance of ~10 mm from the carina of tracheanear, and the percentage of lumen stenosis was greater than 70%. (C) Postoperation, the tumor was removed. The left and right main bronchi, as well as the lobes and segments of the bronchi, were all unobstructed.

Bronchoscopy examination showed a neoplasm located approximately 10 mm from the carina, causing more than 70% luminal stenosis (Figs. 2A–C). Brush biopsy of the neoplasm showed adipocyte hyperplasia.

The patient subsequently underwent a rigid bronchoscopy and lesion resection. Following induction of general anesthesia with intravenous sufentanil and propofol, the patient was pre-oxygenated via face mask with 100% oxygen. After achieving 100% oxygen saturation and adequate muscle relaxation, a 14 mm rigid bronchoscope was inserted orally and connected to mechanical ventilation. The tumor was resected using a combination of high-frequency electrocautery snare and cryoablation. This minimally invasive procedure is instrumental in achieving intraoperative hemostasis. Rigid bronchoscopy achieved complete endoluminal resection without complications. The surgical specimen was submitted for pathological analysis.

**Figure 3.**
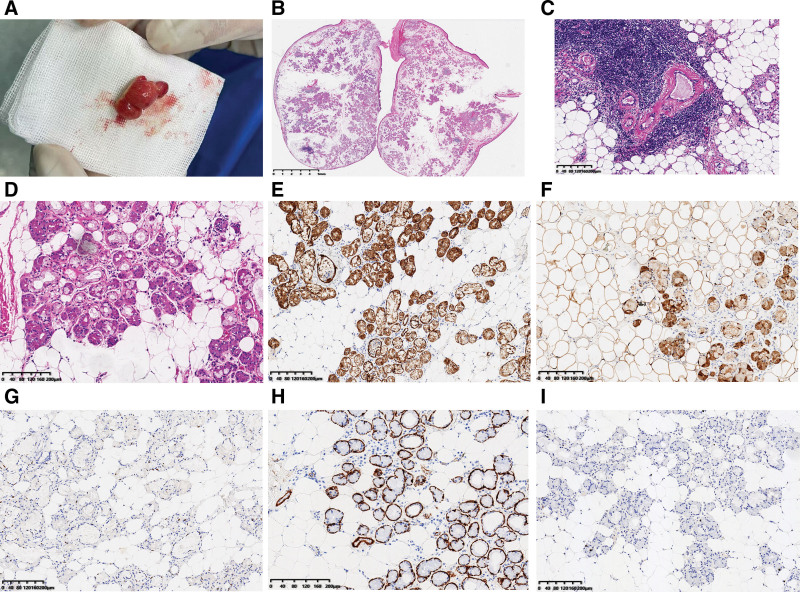
The characteristics of pathology. Gross image, hematoxylin–eosin stain (HE), and immunohistochemistry. (A) The sample had a lobulated nodular shape with smooth surface and yellowish to pale pinkish in color. (B) The tumor was well-delineated, surrounded by a capsule of fibrous tissue, composed of neoplastic mature adipocytes interlaced with salivary gland acini and ducts. (C, D) The ducts exhibited dilatation, hyperplasia and thin fibrous tissue around them. Acinar atrophy and lymphocytic infiltrate could be observed. (E) CK-pan highlighted ductal epithelium. (F) S100 labeled acinar and the adipose cells. (G) Myoepithelium cells were positive for P63. (H) Calponin labeled myoepithelium cells. (I) The Ki-67 proliferation index was low.

Macroscopically, the sample appeared as a lobulated nodule with a smooth surface and yellowish to pale pink coloration, measuring 18 × 12 × 10 mm (Fig. 3A).

Microscopically, the tumor was well-delineated and surrounded by a fibrous capsule, composed of neoplastic mature adipose tissue mixed with nonneoplastic salivary ducts and acini (Figs. 3B–D), consistent to sialolipoma. The adipose tissue constituted about 40% of the tumor mass. The ducts exhibited dilatation, hyperplasia and thin fibrous tissue around them. Acinar atrophy and lymphocytic infiltrate could be observed. Immunohistochemistry showed CK-pan positivity in ductal epithelium (Fig. 3E); S100 positivity in acinar and adipose cells (Fig. 3F); P63 and Calponin were positive in myoepithelium cells (Fig. 3G, H). The proliferative index (Ki-67) was low (Fig. 3I). This result would reinforce benign behavior of the tumor. The patient was diagnosed with sialolipoma.

## 3. Discussion

Sialolipoma is exceedingly rare, with only a few dozen cases reported. It occurs primarily in both major and minor salivary glands, with a predominant incidence in the parotid glands.^[2]^ Studies by Nagao et al have confirmed that sialolipoma contains ducts, acini, basal cells, and myoepithelial cells. The cellular and structural composition closely resembles normal salivary gland tissue.^[1]^ Aside from minor changes such as ductal ectasia with fibrosis and focal oncocytic metaplasia, these components do not demonstrate atypia. The salivary components in sialolipoma are typically nonneoplastic, embedded within a lipomatous tumor. Sialolipoma can occur in individuals of any age, ranging from 0 month to 84 years,^[2]^ with a higher prevalence in adults over the age of 18.^[9]^

Sialolipoma occurring in the tracheobronchial tree are extremely rare, with only 6 cases reported to date, 2 of which were diagnosed as adenolipoma.^[3–8]^ These cases are summarized in Table 1. In the present case, the patient was a young adult (28 years old), with a trachea tumor, and then underwent endotracheal tumor resection.

**Table 1:** Summary of salivary-type lipomatous lesions of the tracheobronchial tree (sialolipoma/adenolipoma).

Case	Diagnosis	Age	Sex	Site	Size (mm)	Treatment	Outcome
Nakata et al^[7]^	Adenolipoma	75	F	Left upper division and lingular bronchus	NA	Left upper lobectomy with mediastinal and hilar lymph nodal sampling	NED at 61 months
Kajiwara et al^[5]^	Sialolipoma	58	M	Left main bronchus	20	Tendotracheal tumor resection	NED at 51 months
Cherian et al^[8]^	Adenolipoma	68	F	Left main stem bronchus	NA	Endotracheal tumor resection with minimal residual disease	Reexpansion of the left upper lobe with a small remnant tumor after 1 year
Nodar et al ^[3]^	Sialolipoma	52	F	Left entrance of the main bronchus	NA	Endotracheal tumor resection	NED
O’Neill et al^[6]^	Sialolipoma	71	F	Left lower lobe bronchus	85	Left lower lobectomy	NA
Parkhi et al^[4]^	Sialolipoma	52	M	Right intermediate bronchus	32	Endotracheal tumor resection	NED

F = female, M = male, NA = not available, NED = no evidence of disease.

* Sialolipoma and adenolipoma essentially refer to the same tumor. Based on the benign nature of the salivary gland components in the tumor, the recent WHO classification (2022) does not recommend using the term “adenolipoma”.

The main diagnostic features of sialolipoma include: A well-circumscribed tumor encapsulated by thin fibrous tissue.^[1]^ A composition of mature neoplastic adipose tissue interspersed with scattered, atrophied nonneoplastic salivary gland acini and ducts.^[1,10]^ The proportion of adipose tissue varied from 40% in the minor salivary glands to over 90% in major salivary glands.^[1,11]^ In this case, the proportion of adipose was 40%. Acinar atrophy, duct dilation, fibrosis, lymphocytic infiltration, and oncocytic metaplasia may occur.^[12]^ In this case, acinar atrophy, duct dilation, fibrosis, and lymphocytic infiltration were observed, but oncocytic metaplasia was not observed.

Endotracheal sialolipoma should be distinguished from the following tumors. Endotracheal lipoma: This tumor appears as a fat density, with a CT value ranging from—40 to–120 HU and shows no enhancement. Histologically, endotracheal lipoma has no salivary elements. Mucous gland adenoma (MGA): MGA was observed as a well-defined nodule on CT with air-meniscus sign.^[13]^ Histologically, MGA is characterized by glandular proliferation without a predominant adipose component. Endobronchial hamartoma: CT scans show rounded soft-tissue masses that frequently contain characteristic “popcorn” calcifications and fat density.^[14]^ This tumor may contain fat. It often includes cartilage/mesenchymal elements. Respiratory epithelial adenomatoid hamartoma: On CT, respiratory epithelial adenomatoid hamartoma typically presents as a well-defined soft-tissue attenuation mass. This tumor exhibits elongated or ovoid glands, descending from the surface. These glands are lined by ciliated epithelium with mucinous cells, and are enveloped by thick, hyalinized stroma. Pleomorphic adenoma with lipometaplasia: CT and MR images reveal various amounts of fat density in the tumor.^[15]^ Histologically, this tumor consists of tumorous glandular epithelium, variant myoepithelium, chondromyxoid stroma, and presents an extensive area of lipomatous components.

The pathogenesis of sialolipoma is not completely understood. Several hypotheses have been proposed: The acinar and ductal components in sialolipoma present elements of a hamartomatous process.^[16]^ Sialolipoma is a distinct variant of salivary gland lipoma.^[1]^ Sialolipoma may be associated with salivary gland dysfunction, leading to morphological changes in salivary tissue.^[11]^

Sialolipoma grows slowly, and early-stage lesions often present with no significant clinical symptoms. When the tumor occurs in the tracheobronchial tree, increasing size can lead to symptoms such as cough, increased sputum, hemoptysis, fever, dyspnea, and wheezing. CT and MRI can assist in diagnosis by demonstrating characteristic features, such as low-intensity CT signal and high MRI intensity. The preferred treatment is endotracheal tumor resection. Prognostically, sialolipoma is a benign tumor with no recurrence or malignancy postoperation in vast majority of patients. Very few cases recur due to incomplete tumor resection. At the 7-month postoperative follow-up, there was no clinical or radiological (CT scan) evidence of recurrence.

The study’s limitations in our work is the insufficient duration of follow-up. The patient was followed for 7 months, and the possibility of recurrence could not be completely excluded. Accurate prognosis requires long-term follow-up.

In conclusion, we described a rare case of endotracheal sialolipoma, detailing its clinical symptoms, imaging and pathological features, treatment, and follow-up outcomes. This tumor can lead to symptoms such as cough, increased sputum, hemoptysis, fever, dyspnea, and wheezing. CT and MRI are valuable diagnostic tools (CT Hounsfield units suggests fatty components in the tumor). Histologic examination revealed a well-circumscribed lesion composed of mature adipose tissue admixed with nonneoplastic salivary gland ducts and acini. Endotracheal sialolipoma is a rare benign tumor. The primary treatment is endoscopic tumor resection. The prognosis is favorable after complete excision.

## Author contributions

**Data curation:** Qiong-Nan Shen.

**Investigation:** Jian-Feng Chu.

**Resources:** Zhi-Bin Gao.

**Writing – original draft:** Ying Xu.

**Writing – review & editing:** Ying Xu.
